# Ecological Momentary Mood, Resilience, and Mental Health Status as Predictors of Quality of Life Among Young Adults Under Stress: A Structural Equation Modeling Analysis

**DOI:** 10.3389/fpsyt.2021.672397

**Published:** 2021-06-22

**Authors:** Rachel-Tzofia Sinvani, Haya Fogel-Grinvald, Anat Afek, Rina Ben-Avraham, Alex Davidov, Noa Berezin Cohen, Ariel Ben Yehuda, Mor Nahum, Yafit Gilboa

**Affiliations:** ^1^School of Occupational Therapy, Faculty of Medicine, The Hebrew University of Jerusalem, Jerusalem, Israel; ^2^Medical Branch, Ground Forces, Israel Defense Forces, Haifa, Israel; ^3^Department of Health and Well-Bring, Medical Crops, Israel Defense Forces, Ramat Gan, Israel

**Keywords:** military training, gender, distress, psychological resilience, ecological validity, combat soldiers

## Abstract

Multiple internal factors, such as psychological resilience and mental health status, have been shown to contribute to overall quality of life (QoL). However, very few studies to date have examined how these factors contribute to QoL of youth and young adults in a stressful situation. Here, we studied the contribution of these factors, as well as of ecological momentary mood assessment, to QoL of young army recruits during their Basic Training Combat (BCT). To this end, we collected data from 156 male and female soldiers in a mixed-gender unit in the Israel Defense Forces (IDF). Using a mobile app installed on participants' phones, participants provided self-reports regarding their mental health status and psychological resilience at baseline, and QoL 2 weeks later. Momentary mood reporting was further collected during the 2-week interval period using a daily self-report mood scale (IMS-12). Structural equation modeling (SEM) was used to examine the interrelationships among the study variables based on a hypothesized model. We found that a model with all factors (gender, resilience, mental health status and momentary mood) provided a good fit for the data based on its fit indices [χ^2^(38) = 47.506, *p* = 0.139, CFI = 0.979, NFI = 0.910, RMSEA = 0.040, TLI = 0.964]. However, the only direct contributors to QoL were gender and momentary mood, accounting together for 61.5% of the variance of QoL. Psychological resilience and mental health status contributed to QoL only indirectly, through their associations with momentary mood. Collectively, these results highlight the importance of ecological momentary assessment of mental-health related factors such as mood to the prediction of QoL in young adults under stress. These findings may have broader implications for monitoring and improvement of well-being in young healthy populations as well as in clinical ones.

## Introduction

Adolescence and early adulthood are among the most critical periods of human development, when the environment and physiological changes have significant impact on one's life (1). Understanding the role of adolescents as the future of society yields tremendous interest in guaranteeing their mental health and quality of life (QoL) as a major concern of all societies (2). Although the vast majority of adolescents are finally satisfied with their lives and generally report good health, an increasing number of adolescents report psychosocial challenges and health complaints in everyday life (3, 4).

In Israel, most youth and young adults begin their mandatory military service in the Israel Defense Forces (IDF) in the ages of 18–19 years (5), when the prevalence of stress-related disorders is high (6). The transition to military life from the civilian environment is stressful on its own and requires individuals to adapt to a strict discipline, extensive physical training, institutional feeding, and separation from friends and family (7–9). Although many Israelis are motivated to serve in IDF and face the challenges related to the military service (10), difficulties in adjusting to the new environmental are frequent (11). While the negative impact of stress on QoL has been well-documented in multiple adolescent populations, such as in those living in high threat environments (12, 13), in at-risk youth (14), in university students (15) and in college students (16), little is known about contributors to the QoL of newly recruited soldiers during their BCT in the army worldwide, as most studies examining QoL were conducted in veterans or in combat soldiers after their basic training phase (17, 18).

QoL is defined as an individuals' position in life in the context of the culture and value systems in which they are inserted, including their goals, expectations, standards, and concerns (19). Among the most cited theoretical models accounting for QoL of different populations during stressful life periods is the conceptual model of Health-Related Quality of Life (HRQoL) (20). The model emphasizes two major factors contributing to overall QoL: intrinsic factors, such as personal factors and psychological characteristics, and extrinsic factors which are environmental characteristics, such as one's workplace and lifestyle (20). The current study aims to account for QoL in newly drafted soldiers during the stressful period of Basic Combat Training (BCT). During BCT, since all soldiers are exposed to the same stressful environment (i.e., the same external factors), there is an opportunity to examine the unique contribution of intrinsic factors. Specifically, we focus here on the intrinsic contributors of psychological resilience, self-efficacy, mental health status, momentary mood and gender for QoL.

Conducting the current study in the mixed gender units of IDF, in which male and female recruits undergo the same training and eventually serve in similar positions, allowed us to further assess the contribution of gender to QoL. Although female recruits have been part of combat units in the military for several years, data regarding their psychological adaptation and psychological resilience in relation to their QoL is still mixed (21, 22). Specifically, while some studies report no differences in the degree of self-reported adversities in females compared to their male counterparts (5, 7, 17), others find higher levels of self-reported adversities among newly-recruited females soldiers (23–25). These excessive mental challenges can lead to severe drops in the various domains of QoL in female compared to male soldiers (26, 27), as was indeed found in a few recent studies (28, 29).

A significant contributor to QoL in adolescence is mental health status, which refers to the experience vs. absence of some mental disorders such as anxiety disorders, trauma- and stressor-related disorders, as well as personality disorders (30). The prevalence of mental health disorders among young individuals has been reported to be high across different cultures, genders, and age ranges (31, 32). Moreover, when experienced during adolescence, mental health challenges can have a long-term impact with significant consequences on QoL (33, 34). In line with previous models, the negative impact of mental challenges is regarded as a risk factor for perceived QoL among adolescents (14, 35). Interestingly, there is no consensus in the literature regarding gender differences in mental health problems in adolescence: while some studies reported that the incidence of mental disorders as a result of stressful conditions is higher among female compared to male adolescents, other studies found no such gender differences (14, 17, 36, 37).

Mental health status is further characterized by high intra-individual variability, as well as by high inter-individual changes over time (38). However, standard assessment methods, which are mainly based on retrospective self-reports and subjective clinical impression, are limited in their ability to accurately characterize day-to-day variations in those symptoms (39). Due to the experience of symptoms outside the clinical setting or between treatment sessions, monitoring of symptoms as more frequently “in real world” is actually needed (40). Using Ecological Momentary Assessment (EMA), in which data are collected in the natural environment and repeatedly across multiple time points, one can effectively assess the dynamics of mental health-related symptoms in everyday life (41, 42). Indeed, large variability in daily mood reporting (i.e., less mental health stability) was found as a significant predictor of mental health status (40, 41, 43–45). In addition, female adolescents show greater variability in EMA of mood compared with male adolescents (46, 47). Recent studies have further shown that daily positive affect is associated with higher QoL and lower depressive and anxiety symptoms, through the enhancement of psychological resilience (48, 49).

Psychological resilience was further suggested as a protective factor which may positively contribute to QoL (16). Psychological resilience can be considered as either a trait, representing a constellation of characteristics that enable individuals to adapt to the circumstances they encounter, or as a state, a dynamic process encompassing positive adaptation within the context of significant adversity (50). Self-efficacy, the strong belief in one's ability to achieve designated aims or accomplish specific tasks (51), is another protective factor conceptually related to psychological resilience. Individuals with high degree of self-efficacy might view stressors as an opportunity, rather than a challenge. Furthermore, they might be more capable of dealing with certain stressors in life by engaging in active problem-solving strategies (52). Psychological resilience and self-efficacy are closely related and even slight overlap (53), and it is unlikely that they each affect well-being independently from one another (54). Therefore, in our model, we refer to self-efficacy as part of the global concept of psychological resilience. In addition, there may be gender differences related to the two constructs: while female adolescents were found to report lower self-efficacy under stress compared to their male counterparts (55, 56), others report higher levels of challenges among newly-recruited females soldiers (24).

**Figure 1:**
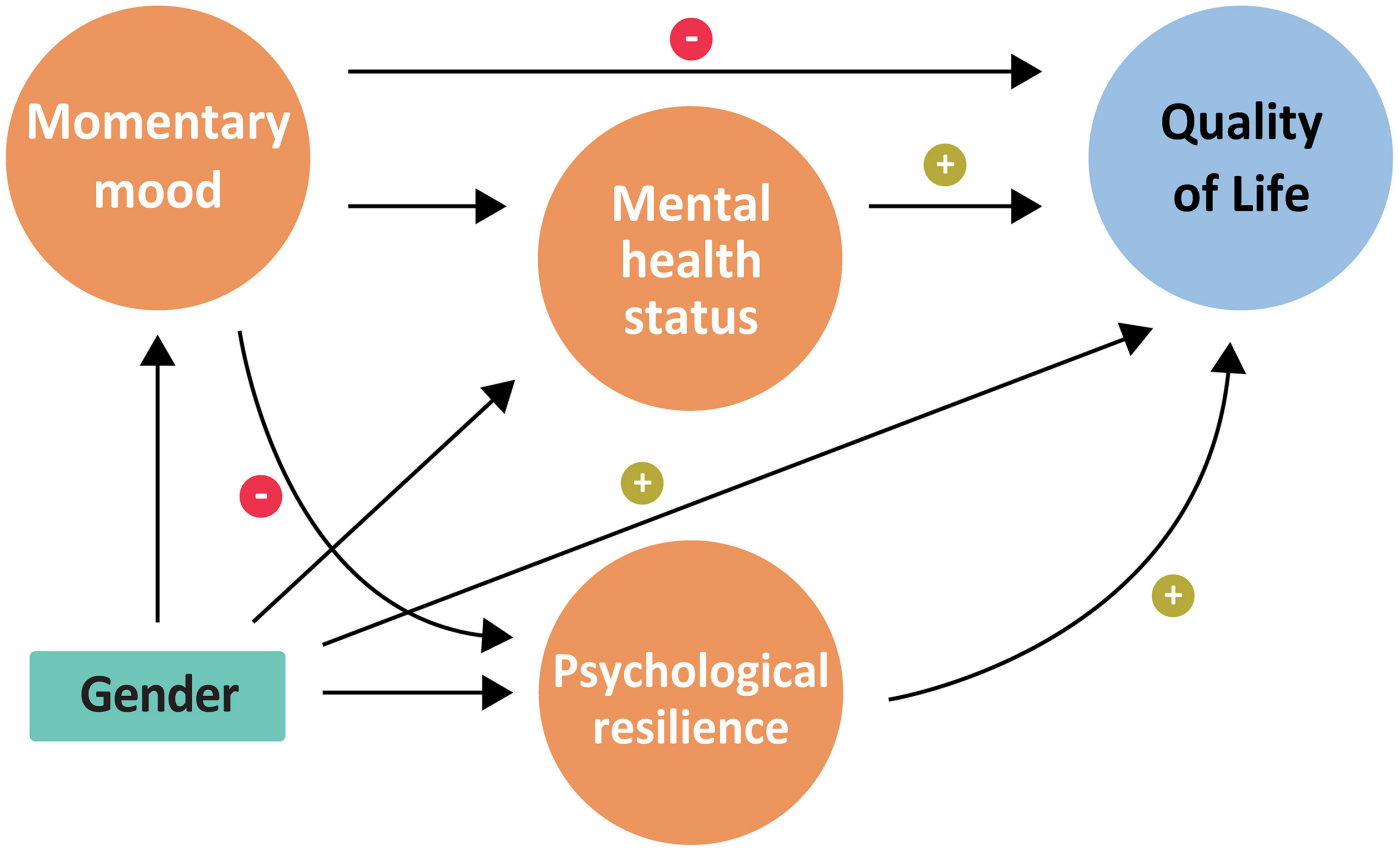
A theoretical framework to account for QoL from gender, psychological resilience, mental health and momentary mood.

In the current study, we aimed to examine, for the first time, the contribution of psychological resilience, mental health status and momentary mood to QoL in female and male recruits during BCT. We used Structural Equation Modeling (SEM) (57) in order to test the factors which potentially contribute—directly or indirectly—to the QoL of male and female soldiers along their BCT. On the basis of the literature cited above and of theoretical guidelines related to the HRQoL model (20) we hypothesized that psychological resilience will be directly and positively associated with QoL, and also indirectly, *via* its contribution to mental health status (16, 58). We further predicted that mental health status will have a positive association with QoL (59, 60). Momentary mood is expected to affect QoL both directly and indirectly, mediated by psychological resilience (61). Finally, in line with the predicted effects of gender on QoL (56), we hypothesized that gender will directly contribute to QoL. Additionally, following the ongoing debate regarding the impact of gender on mental health status and on psychological resilience, we hypothesized an additional direct contribution of gender to both variables (37). The theoretical model we examined is depicted in Figure 1.

## Materials and Methods

### Participants

A convenience sample of 156 IDF soldiers was recruited for the study. All participants were healthy young adults that meet the health requirements of IDF for inclusion in a combat unit (62). We excluded from this report one participant whose gender was not recorded. Participants were from two recruiting cycles of the border defense infantry battalions, during their BCT, between April 2018 and October 2019. Data collection underwent at the recruit's military base in the Southern part of Israel. The border defense infantry battalions include both male and female recruits who undergo similar training together. Participants were included in the study if they were 18 years of age at time of consent and owned a smart mobile phone which can be used in the study. Participants did not receive monetary compensation for their participation.

### Study Procedures

The study was approved by the IDF medical corps Institutional Review Board (IRB). All participants gave written informed consent before engaging in any study-related activities. Following informed consent, participant completed baseline assessments (t0) and then completed 2 weeks of EMA. At the end of the 2-week tracking period, participants repeated the assessment battery (t1). In the current study, we include the results of the self-report data collected during t0 and the QoL data collected during t1, as well as the mood data collected during the 2 weeks of the tracking period. Data from other aspects of the trial are reported elsewhere (63).

### Study Materials

We used the Hebrew versions of self-report validated questionnaires to assess QoL (at t1), mental health, psychological resilience and self-efficacy. The overall completion time for the entire battery at t0 lasted ~15 min.

#### Quality of Life

The World Health Organization's QoL Instrument-abbreviated version [WHOQOL-BREF; (64), Hebrew version]. The WHOQOL-BREF instrument is a self-administered questionnaire, comprised of 26 items which collectively assess the four major QoL domains defined by the WHO: physical health, psychological health, social relations, and environment. The fourth domain is composed of environmental-related items (e.g., leisure activities, living place and transport), which are less relevant in the context of BCT. We therefore used only the first three domains in the current study. Each question is rated on a 5-point Likert scale, and scores of all domains are summed and scaled in a positive direction such that higher scores indicating better QoL (64). The WHOQOL-BREF has good to excellent psychometric properties of reliability and performs well in preliminary tests of validity. It has further been found as a valid tool for quality-of-life assessment in similar samples, such as police officers and soldiers (65, 66). In our sample, the entire scale as well as the subdomains have good internal consistency (Cronbach's α for entire scale = 0.846; sub-domains: 0.648–0.726), similar to that found in previous studies (Cronbach's α = 0.867; sub-domains: 0.755–0.793) (67).

#### Mental Health

##### Psychological Distress

The Kessler Psychological Distress Scale - 6-item [K6; (68)]. The purpose of this self-report questionnaire is to measure the subjects' level of distress. The scale is comprised of six statements; all are related to the frequency of which the participant experienced distress in the last 30 days. Items are rated on a 5-point Likert scale, ranging between 0 (never) and 4 (always). The final score ranges between 0 and 24, with higher scores indicating more distress (69). The scale has high internal consistency in our sample (Cronbach's α = 0.806). Previous studies found similarly high internal consistency (0.89), as well as good sensitivity (SE = 0.36) and specificity (0.96) in predicting severe mental illness (68).

##### Anxiety

Generalized Anxiety Disorder, 7-item [GAD-7; (70)]. GAD-7 is a standardized, validated self-report questionnaire used to assess anxiety. It includes 7 items describing the severity of the subjects' anxiety over the past 2 weeks on a 4-point Likert scale (0 = not at all sure, 3 = nearly every day). The sum score ranges from 0 to 21, with higher scores indicate more severe anxiety symptoms. The scale has high internal consistency in our sample (Cronbach's α = 0.848). A factor analysis further confirmed that the items in the GAD-7 are distinct from those of depression (70).

##### Rumination

Ruminative Response Scale [RRS; (71)]. A standardized, validated 22-item self-report measure of rumination experienced in the 2 weeks preceding administration. Participants were asked to rate the frequency with which they respond to negative mood as described in each item using a 4-point Likert scale, ranging from 1 (almost never) to 4 (almost always). RRS in our sample has high internal consistency (Cronbach's α = 0.904), which is similar to the internal consistency found in previous studies (0.9) (72).

#### Ecological Momentary Assessment of Mood

Immediate Mood Scale [IMS-12; (40)]. A 12-item measure developed to assess the dynamic components of mood. In the current study, we used the IMS-12 scale, delivered on the participant's mobile phones, in order to measure momentary mood twice/daily during the 2-week tracking period (between t0 and t1). Since the soldiers participating in the study did not have their mobile phones with them, direct commanders were asked to provide them with their phones twice/day, once in the morning and once in the evening, to allow them to fill out the IMS-12 scale. Due to their varying schedule during BCT, we provided a broad time window of 4.5 h in the morning (between 6 a.m. and 10:30 a.m.) and in the evening (between 6 p.m. and 10:30 p.m.). The questionnaire could have been filled out only once during each time window and was unavailable in times outside these two morning and evening time windows. Daily reminders were sent to the direct commanders by the study staff, reminding them to give the soldiers who participated in the study their phones during the relevant time window. Participants were contacted by study staff in case they missed several consecutive assessments, to help with any technical issues they encountered.

The IMS-12 scale prompts participants to rate their *current* mood state on a continuum using 12 items (e.g., happy-sad, distracted-focused, sleep-alert, fearful-fearless), each with a 7-point Likert scale. For each item, an integer score between 1 and 7 was derived. The total score for this scale is the sum of the scores on all 12 items. To be consistent with other scales assessing mental health status (e.g., PHQ-9, GAD-7), total score is multiplied by −1, such that where higher scores reflect worse (i.e., more negative) mood states. The scale has been recently used to help identify an amygdala-hippocampus sub-network that encodes variations in human mood (73). We derived the average and standard deviation from the daily mood reporting for each participant.

#### Psychological Resilience

The self-assessed resilience scale (74): a 5-item self-report measure of resilience to stress. Participant were asked to rate their ability to cope with stress on a 4-point Likert scale on each of the 5 items (0 = low ability, 4 = excellent ability). Participants rated their ability to “keep calm and think of the right thing to do in a crisis,” “manage stress,” “try new approaches if old ones don't work,” “get along with people when you have to,” and “keep your sense of humor in tense situations” as poor, fair, good, very good, or excellent. The total score, which is the sum of scores of all five items, ranges between 0 and 20, with higher scores reflecting better psychological resilience. The internal consistency of the scale in our sample is acceptable (Cronbach's α = 0.732). Previous studies found slightly higher internal consistency for this scale (Cronbach's α of 0.86–0.89) (75).

#### Self-Efficacy

The New General Self-Efficacy Scale (NGSE) (76). A 10-item standardized questionnaire capturing one's belief regarding one's ability to perform specific leadership behaviors successfully. Each item is rated on a 4-point Likert scale (from 1 = strongly agree to 4 = strongly disagree). The total score ranges from 0 to 40, with higher scores indicating higher levels of self-efficacy. Internal consistency of the scale for our sample is high (Cronbach's α = 0.916), which is similar to that reported in other studies (α = 0.86) (76).

### Data Analysis

Statistical analysis was performed using IBM SPSS (Statistical Package for the Social Sciences) version 25.0 (77) and IBM AMOS Graphics software version 25.0 (78). Descriptive statistics were used to examine the demographic characteristics, and the questionnaires. A one-sample *t*-test was used to compare questionnaire data from our study to that derived from norms obtained from young healthy populations Independent *t*-tests were conducted to examine gender differences across all measures (79). Correlations between variables indicate the level of difference and the discriminant validity of the variables. Confirmatory factor analyses (CFA) for the Structured Equation Model (SEM) were conducted in AMOS. SEM with maximum likelihood estimation was used to test the hypothesized model. Model fit was assessed using the following goodness-of-fit indices: chi-square, Comparative Fit Index (CFI), Tucker-Lewis Index (TLI), and Root-Mean-Square Error of Approximation (RMSEA). A non-significant chi square, CFI and TLI ≥ 0.95, and RMSEA ≤ 0.06 (80) are indicative of an acceptable fit. The standardized path coefficients were assessed to examine the statistical significance and directions of path estimates that exist between the variables in the model. For all analyses, *p* < 0.05 was considered statistically significant.

## Results

### Characterization of Study Sample

A total of 156 participants from an IDF combat unit, 98 females (63%) and 58 males (37%), completed the study (age range: 18.1–21.6 years, mean: 19.05 ± 0.57 years). Table 1 lists the demographic variables and outcome measures in the study sample by gender. Average scores of the QoL subscales (WHOQOL-BREF) for the study sample were significantly lower compared to the general young population for all three domains [Domain 1: 52.4 ± 17.6 vs. 76.5 ± 12.6, *t*_(144)_ = −16.439, *p* < 0.001; Domain 2: 65.0 ± 17.1 vs. 67.7 ± 15.7, *t*_(144)_ = −1.894, *p* < 0.05; Domain 3: 64.6 ± 23.2 vs. 69.4 ± 19.2, *t*_(144)_ = −2.451, *p* < 0.01 in study sample compared to normative data, respectively; (81)]. Similarly, mental health status, as was measured by psychological distress and anxiety, was significantly worse (i.e., higher average scores) compared to norms obtained from the general healthy population [K6: *t*_(155)_ = 10.097, *p* < 0.001; GAD-7: *t*_(151)_ = 14.329, *p* < 0.001] (82, 83).

**Table 1:** Descriptive statistics of demographic variables and outcome measures in the study sample.

		**Total (*****n*** **=** **156)**		**Females (*****n*** **=** **98)**		**Males (*****n*** **=** **58)**		
		**Range**	**Mean (SD)**	**Range**	**Mean (SD)**	**Range**	**Mean (SD)**	***t*_**(154)**_**
Age		18.10–21.59	19.06 (0.59)	18.13–21.59	19.06 (0.60)	18.10–20.56	19.08 (0.58)	0.182
Quality of life (t1)	WHOQOL-BREF-Dom 1	6–94	52.41 (17.65)	6–94	50.51 (18.18)	25–88	55.70 (16.32)	1.716
	WHOQOL-BREF-Dom 2	19–100	65.01 (17.12)	19–100	61.72 (17.82)	44–100	70.72 (14.25)	3.141^**^
	WHOQOL-BREF-Dom 3	0–100	64.66 (23.28)	0–100	63.80 (24.19)	25–100	66.15 (21.75)	0.583
Mental health status (t0)	Psychological distress (K6)	0–24	9.49 (4.69)	0–24	10.12 (4.78)	1–18	8.43 (4.38)	−2.203^*^
	Anxiety (GAD-7)	0–21	8.74 (4.98)	0–21	9.29 (5.10)	0–19	7.76 (4.65)	−1.829
	Rumination (RRS)	22–79	46.59 (12.41)	23–79	47.65 (12.74)	22–68	44.75 (11.71)	−1.396
Momentary mood (t0)	IMS-12 - mean	(−91)–(−39)	−66.51 (11.13)	(−87)– (−41)	−65.29 (11.10)	(−91)–(−39)	−68.49 (10.99)	−1.733
	IMS-12 - SD	1–32	12.19 (6.71)	2–32	13.99 (7.12)	1–20	9.24 (4.72)	−4.470^***^
Resilience (t0)	Stress-resilience	2–20	13.36 (3.76)	2–20	13.09 (3.76)	2–20	13.80 (3.76)	1.123
	The new general self-efficacy scale (NGSE)	8–40	31.52 (5.78)	8–40	31.23 (5.51)	11–40	32.02 (6.24)	0.808

* *p < 0.05*;

** *p < 0.01*;

*** *p < 0.001*.

Finally, participants filled out, on average, 7.5 ± 3 times the mood EMA (IMS-12) during the 2-week tracking period between t0 and t1. There were no gender differences in adherence to the EMA protocol [7.59 ± 2.96 vs. 7.48 ± 3.16 sessions for female and male groups, on average; *t*_(151)_ = −0.21; *p* = 0.83]. Altogether, 1,170 samples of momentary mood were obtained from study participants.

### Gender Differences in Psychological Resilience, Mental Health Status, Momentary Mood, and QoL

We compared scores on the self-report scales across genders. For QoL, gender differences were found for psychological QoL (domain 2) only, with female paticipants reporting overall lower psychological QoL compared to male participants [61.7 ± 17.8 and 70.7 ± 14.2 for female and male participants, respectively; *t*_(154)_ = 3.14, *p* < 0.01; see Table 1]. No gender differences were found for psychological resilience or for the mental health scales of anxiety and rumination. However, female participants reported higher levels of psychological distress compared to male participants [10.2 ± 4.8 and 8.4 ± 4.3 for female and male participants, respectively; *t*_(154)_ = −2.22, *p* < 0.05; see Table 1]. Finally, while the average score of the momentary mood reporting did not differ between genders, variability in mood reporting (IMS-12 SD) was higher for female compared to male participants [13.9 ± 7.1 vs. 9.24 ± 4.7 for female and male participants respectively; *t*_(154)_ = −4.47, *p* < 0.001; see Table 1].

### Correlations Among Outcome Measures

Table 2 describes the correlations between the study outcome measures. QoL was positively correlated with psychological resilience and with self-efficacy and was significantly negatively correlated with the mental health components (psychological distress, anxiety and rumination).

**Table 2:** Pearson correlational analysis of the relationships between study variables.

		**1,r**	**2,r**	**3,r**	**4,r**	**5,r**	**6,r**	**7,r**	**8,r**	**9,r**
QoL (t1)	1. WHOQOL-BREF-Dom1	1								
	2. WHOQOL-BREF-Dom2	0.505^***^	1							
	3. WHOQOL-BREF-Dom3	0.205^*^	0.495^***^	1						
Mental Health	4. Distress (K6)	−0.354^***^	−0.355^***^	−0.196^*^	1					
	5. Anxiety (GAD-7)	−0.298^***^	−0.369^***^	−0.171^*^	0.64^***^	1				
	6. Rumination (RRS)	−0.26^**^	−0.405^***^	−0.286^**^	0.456^***^	0.612^***^	1			
Momentary	7. IMS-12 Mean	−0.352^***^	−0.446^***^	−0.330^***^	0.481^***^	0.405^***^	0.353^***^	1		
Mood	8. IMS-12 SD	−0.426^***^	−0.467^***^	−0.079	0.270^***^	0.281^***^	0.214^**^	0.339^***^	1	
Resilience	9. Stress-Resilience	0.274^**^	0.405^***^	0.209^*^	−0.332^***^	−0.379^***^	−0.300^***^	−0.405^***^	−0.234^**^	1
	10. NGSE	0.199^*^	0.358^***^	0.214^*^	−0.278^***^	−0.248^**^	−0.230^**^	−0.363^***^	−0.179^*^	0.559^***^

* *p < 0.05*;

** *p < 0.01*;

*** *p < 0.001*.

As expected, significant positive correlations were found between psychological resilience and self-efficacy, such that higher levels of psychological resilience were associated with higher levels of self-efficacy. Psychological resilience and self-efficacy each showed significant negative correlations with the mental health components, such that higher levels of psychological resilience and of self-efficacy were associated with better mental health. In addition, the correlations within the mental health variables themselves (psychological distress, anxiety, and rumination) were significant and positive.

Finally, the average and variability of the daily momentary mood (IMS-12 average and SD) were positively correlated with mental health status, and negatively correlated with psychological resilience, self-efficacy and QoL. In other words, those with higher average mood and more variable mood had lower levels of psychological resilience, of self-efficacy and of QoL, and worse mental health status.

### Structural Equation Model Analysis

**Figure 2:**
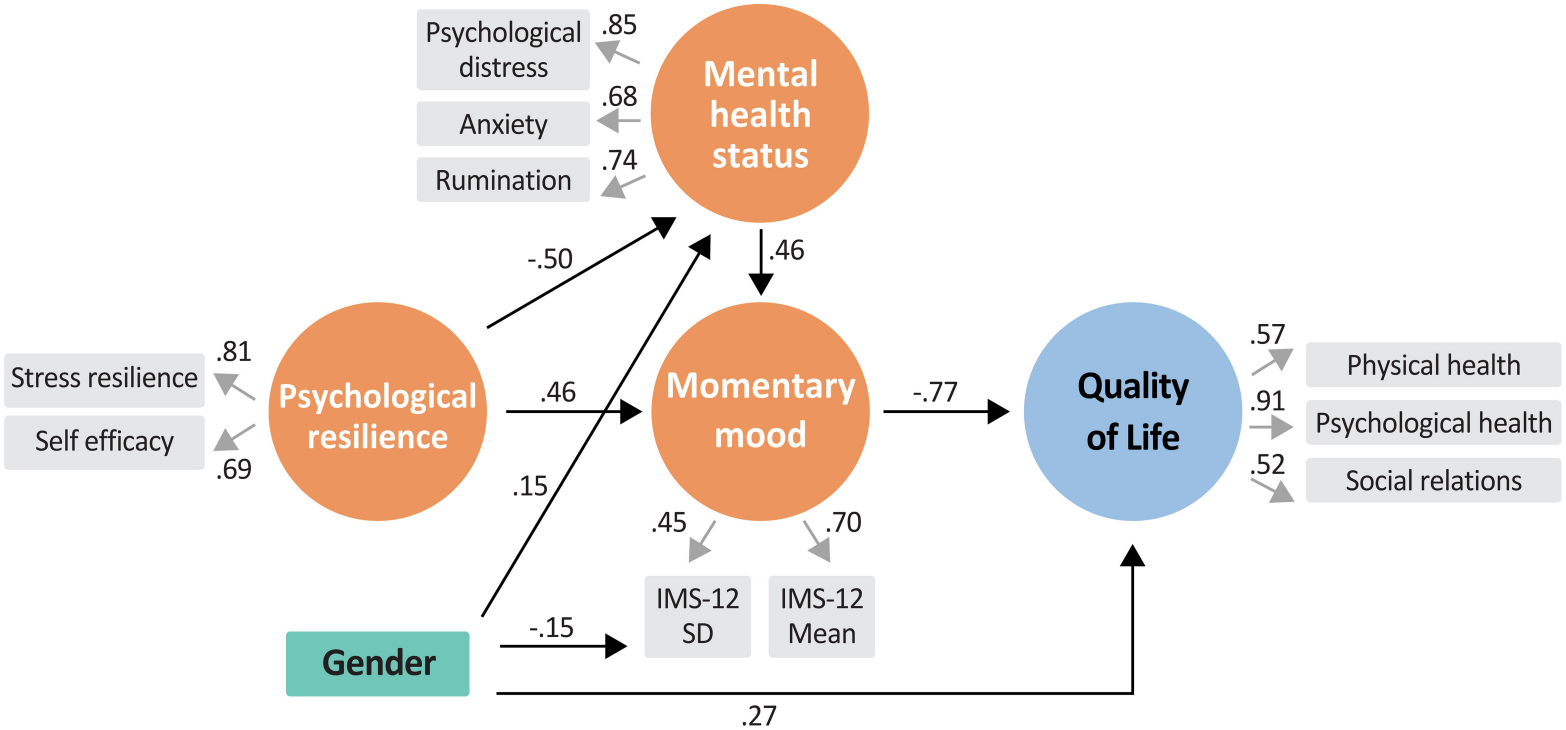
A Structural equation model (SEM) analysis of the effect of psychological resilience, mental health and momentary mood on quality of life. χ^2^(38) = 47.506, *p* = 0.139, CFI = 0.979, NFI = 0.910, RMSEA = 0.040, and TLI = 0.964.

We performed a path analysis in order to test the potential effect of psychological resilience, mental health status, self-efficacy and momentary mood on QoL. The results are presented in Figure 2 and Table 3. Four latent constructs (psychological resilience, mental health, momentary mood and QoL) and 11 observed variables were included in the model. All fit indices for the model indicated that it has suitable fit to the data [χ^2^(38) = 47.506, *p* = 0.139, CFI = 0.979, NFI = 0.910, RMSEA = 0.040, and TLI = 0.964].

**Table 3:** Direct, indirect and total effects of the structural equation model (SEM).

**Dependent variable**	**Independent variable**	**Direct effect β (*p*)**	**Indirect effect β (*p*)**	**Total β (*p*)**
Mental health	Gender	0.146 (0.074)	–	0.146 (0.074)
	Resilience	−0.505 (<0.001)	–	−0.505 (<0.001)
IMS-12 SD	Gender	0.273 (<0.001)	0.032 (0.222)	0.305 (0.010)
Mood	Gender	–	0.067 (0.222)	0.067 (0.222)
	Resilience	−0.454 (<0.001)	−0.234 (0.018)	−0.688 (0.010)
	Mental health	0.463 (<0.001)	–	0.463 (<0.001)
QoL	Gender	−0.145 (0.055)	−0.052 (0.222)	−0.197 (0.010)
	Mood	−0.767 (<0.001)	–	−0.767 (<0.001)
	Resilience	–	0.528 (0.010)	0.528 (0.010)
	Mental health	–	−0.355 (0.016)	−0.355 (0.016)

The only direct contributors to QoL were momentary mood and gender. Momentary mood had a strong direct negative effect on QoL, such that lower mood average and less variable mood were associated with better QoL. The direct negative association between gender and QoL indicates that female participants had overall lower QoL compared with male participants. Gender also indirectly contributed to QoL, *via* mental health and *via* momentary mood variability (IMS-12 SD). The indirect effect of gender on QoL *via* mental health status indicates that females' levels of mental health were lower compared to those of the male participants. The positive association between gender and momentary mood variability indicates that female participants had higher mood variability which in turn was associated with lower QoL.

Psychological resilience did not have a direct effect over QoL. Instead, it affected QoL indirectly *via* its associations with mental health status and momentary mood. Psychological resilience had a direct effect on mental health status (better psychological resilience indicated better mental health status) which in turn affected momentary mood and QoL. Psychological resilience was also associated with better momentary mood average and less mood variability, which in turn contributed to better QoL. Finally, mental health status had an indirect effect on QoL, such that better mental health status was associated with better momentary mood average and less mood variability, which in turn strongly contributed to QoL.

Finally, given the relatively low number of mood reporting sessions collected during the trial, we re-ran the SEM analysis on the more adherent participants. We therefore used data from 136 participants that had at least 5 mood EMA observations, excluding the 21 participants that had <5 observations. The resulting model was the same as the original one [χ^2^(38) = 46.71, *p* = 0.157, CFI = 0.977, NFI = 0.89, RMSEA = 0.042, and TLI = 0.96].

## Discussion

In the current study, we examined the contribution of the intrinsic factors of psychological resilience, self-efficacy, mental health status and ecological momentary mood to QoL in a group of young adults during their BCT. Using SEM, we found that momentary mood and gender were the only direct contributos to QoL. Other variables—psychological resilience, self efficacy and mental health status—contributed to QoL only indirectly, *via* the mediation of momentary mood. Finally, mental health status partially mediated the effect of psychological resilience on QoL. To the best of our knowledge, this is the first study to examine this set of parameters in a single model accounting for QoL in healthy youth and young adults during a stressful life situation. The combined use of single-time measurements along with repeated EMA measures in an ecological setting is a unique characetisric of this study. In addition, the fact that our sample included female and male soldiers during their BCT, where the immediate enviromental conditions are identical for all participants, allowed us to measure the sole contribution of intrinsic factors to QoL. Below we discuss the potential significance of these effects and their contribution to our understanding of QoL during stressful life periods.

### A Contextual View of Psychological Predictors of QoL

A main finding of our study was the surprising lack of *direct* association between psychological resilience and QoL, and between mental health status and QoL. Specifically, both resilience and mental health were indirectly associated with QoL *via* the mediation of momentary mood. These results are in contrast with our preliminary model, which predicted direct and strong associations between these two predictors and QoL, and to several previous studies involving populations of young recruits to a military service (2, 16, 55) or young adults under stress (84), which did find a direct association between these contributors and QoL. Another study, conducted in a sample of 149 medical students, found that anxiety and depression were associated with significantly poorer QOL.

One potential account for the lack of direct contribution of psychological resilience to QoL could be the the operationlization of QOL and reslilince in this study. In the current study, we used the WHOQOL-brief subscales, which collectively measure physical, psychological and social well-being over the past 2 weeks, reflecting one's actual circumstances and experiences rather than their more stable personality characteristics. The term QoL is often used interchangeably with the term well-being, which reflects more stable personality traits (85). The self-assessed psychological resilience scale used here was derived from studies focusing on trait orientation or personality characteristics of resilience, which emphasize the general capacity to successfully cope with adversity (86). This may account for the fact that trait-like psychological resilience only indirectly contributed to the state-like QoL (87). Indeed, previous studies which have found a direct association between resilience and state-like QoL used the state definition of resilience (88, 89), while studies that used the trait-like definition of resilience, as was the case in our study, did not find such a direct association (90, 91).

Interestingly, mental health status mediated the contribution of psychological resilience to QoL in the current study, suggesting that the relationship between better psychological resilience and better QoL is mediated by lower levels of anxiety, reduced rummination and reduced psycological distress. This result is in line with some of the previous studies, showing an indirect effect of resilience on QoL, mediated *via* mental health factors such as anxiety and post-traumatic growth (92). For example, a descriptive correlational study incuding 30 patients with type 1 diabetes found that the association between resilience and general well-being was mediated by anxiety (91). In another study social support played a partial mediating role in the relationship between trait-resilience and QoL among 98 patients with breast cancer (91). Follow up studies should clearly dissociate between state and trait resilience to allow for better understanding of the unique contribution of each one to QoL. This result may shed further light on the mechanisms which potentially mediate and drive positive QoL. Future research should assess ways to improve resilience and other predetermining factors of mental health impact on QoL.

### Momentary Mood as a Significant Contributor to QoL Prediction

The indirect association of both psychological resilience and mental health status to QoL in our study was mediated *via* the momentary mood assessment. Indeed, the most probable account for the lack of direct association between mental health status (and psychological resilience) and QoL is the inclusion of momentary mood assessment in our model, which was the strongest predictor for QoL. To the best of our knowledge, our model is the first to test the unique contribution of momentary mood to QoL together with additional potential contributors. It could be that the robust finding regarding the mediating role of momentary mood in our model is due to the absence of daily mood reporting in previous models accounting for QoL to date (48, 93). The fact that momentray mood was such a strong predictor of QoL may be accounted for by the high ecological validity of repeated momentary mood assesment, which assesses mood in the current moment and in a real world setting (39, 42). However, while the powerful predictive role of ecological momentary mood assessment is well-documented in the context of mental health as they found to be in a high positive correlation (40, 94), little is known about its potential contribution to psychological resilience and to QoL.

Momentary mood was represented in the model *via* both its average and variability over the 2 week period. Interestingly, it was not just the overall positivity or negativity of mood that contributed to the QoL preduction; instead, variability in mood reporting over time played a crucial role in QoL prediction. Thus, those with better average mood and less variable mood reportings over time had overall better QoL. Moreover, less variable mood reporting in the 2 weeks preceding is in fact a better predictor than other variables such as psychological resilience and mental health. This result is in line with some of the previous reports in the literature, showing that mood fluctuations over time are contributorsof psychological health (95) and that mood fluctuations are frequent in response to stressful events (96). Indeed, previous studies have shown that high emotional variability is strongly correlated with symptoms in many mental illnesses (97) and to QoL, irrespective of worse overall mood status (95, 98). Higher variability in momentary mood—i.e., larger emotional shifts over time—may reflect high emotional reactivity to ongoing events, combined with a lack of regulatory control that prevents the emotions from recovering and returning to baseline and hence contributing to reduced QoL (95). As such, adolescents who show high levels of mood variability may be more vulnerable to the development of internalizing behavioral problems (99). Follow up studies should attempt to further scrutinize the unique contribution of ecological momentary mood variation to QoL in youth and young adults under stress.

### Gender Differences and Their Contribution to QoL Prediction

In addition to momentary mood, the only other factor directly associated with QoL in our study was gender, Female soldiers had, lower psychological QoL and greater psychological distress in comparison to their male counterparts. These gender differences are consistent with multiple previous reports showing higher distress levels in young females compared to male peers (100–102). There are multiple possible accounts for this difference in distress and QoL. First, the gender differences may be related to the nature of stressors faced by women in combat training, which may negatively impact their mental health (37). Second, although there are many more women in combat roles in the army in recent years, the adjustments made to combat training regimes, which were historically undertaken by males only, are minimal and may not suffice (103). Finally, gender inequality, which may be a significant stressor for female combatants, can influence their psychological distress and affect their adjustment efforts [see (37)].

In addition, although average mood reportings over time were similar across genders, female participants had overall higher variability in their mood reporting in the 2 week tracking period compared to male participants. This finding is also in line with previous reports showing higher variability in ecological self reports of happiness and sadness in females compared to males (46, 47). Morover, higher mood variability in feamales was linked to higher emotional reactivity to positive and negative interpersonal events as reported on daily checklist for 2 weeks (104, 105), as well as to increased rumination (106). It has been suggested that hormonal changes during adolescence may lead to higher emotional reactivity and more unstable moods in females (107). However, althought the current as well as other previous studies concluded that mood variability is higher in adolescent females (106), they do not take into account moment-by-moment changes in mood, which have also been pointed out as an important ecological mood metric [see (108) for further discussion]. Future studies should take into account additional ecological parameters which may account for QoL.

Interestingly, in the current study we found no significant differneces in psychological resilience between genders. Results from previous literature are mixed in this regard (7, 17, 37), with some showing higher resilience in male soldiers (109) and others finding that female soldiers are more resilient due to their increased self-compassion and empathy (110). Our findings are consistent with studies reporting no gender on psychological resilience, hence showing no greater vulnerability in female soldiers under stressful conditions compared to their male counterparts (17). Specifically, these lack of gender differences in resilience in our sample may be associated with the high motivation of female recruits in the mixed-gender units in IDF, for which female soldiers may volunteer but service is mandatory for male soldiers [see elaboration in (63)]. Thus, the Israeli female soldiers who serve in these units are possibly more motivated to serve in a combat occupation and environment, and hence show relatively high levels of general predisposition trait resilience.

### Study Limitations

The present study has several limitations that should be noted. First, our sample included adolescents and young adults from a distinct mixed-gender unit of the IDF. The relative homogeneity of the sample in terms of age and nationality and the relatively low number of male vs. female participants may limit the generalizability of the results, and calls for replication studies in other samples. Second, models accounting for QoL take into account additional protecting factors, such as socioeconomic status and social support, that were missing from our study. These need to be further explored in follow up studies. In addition, our outcome measures mainly included self-report questionnaires, which are known to be biased, especially when retrospectively reporting mental health status (40, 41, 44). Studying the association between measures of self-report, without more objective data indicators, has only limited implications. Future studies should consider using more objective metrics to assess resilience, mental health and QoL. Another limitation is related to the relatively low number of EMA observations completed by the group, due to the multiple reasons mentioned above. This number is low compared to other studies (43, 111) and may limit the generalizability of the results. Finally, the mood reporting scale in our study did not allow for separate analysis of negative vs. positive mood – which seems important based on previous literature. Future studies should include additional tools that may be sensitive to such distinction.

### Implications for Future Studies

The results of our study provide support for the centeral role of momentary mood in mediating the link between trait-resilience and mental health, to QoL among youth in a stressful situation. These results emphasize the importance of considering the inclusion smartphone-delivered EMA tools in QOL models. Motivated by recent technological advances, EMA have seen a rise in behavioral medicine research that in real-time, provides the context for behavior in a natural setting. In terms of practical implications, our results support incorporating ecological momentary mood based interventions as part of an intervention suite for improving QoL among youth. Indeed, Ecological Momentary Intervention (EMI) may bridge the gap in current youth mental health care by enableing better access to interventions in a given moment and appropriate context in daily life (112). Thus, novel interventions may incorporate EMI with existing interventions in order to achieve better QoL amidst stress in youth and young adults.

## Data Availability Statement

The raw data supporting the conclusions of this article will be made available by the authors, without undue reservation.

## Ethics Statement

The studies involving human participants were reviewed and approved by the Institutional Review Board (IRB) of the medical corps of Israel Defense Forces (IDF). The patients/participants provided their written informed consent to participate in this study.

## Author Contributions

R-TS wrote the initial draft of the manuscript. HF-G helped with data analysis. AA and RB-A contributed to study design, data collection, and initial data analysis. AD and NC contributed to data collection and study conceptualization. AB contributed to project conceptualization, methodology, supervision, and funding acquisition. YG and MN were in charge of conceptualization of the project, methodology, writing, supervision, project administration, and funding acquisition. All authors reviewed and approved the final manuscript.

## Conflict of Interest

The authors declare that the research was conducted in the absence of any commercial or financial relationships that could be construed as a potential conflict of interest.
